# Nutritional Value of Canteen Menus and Dietary Habits and Intakes of University Students in Indonesia

**DOI:** 10.3390/nu14091911

**Published:** 2022-05-02

**Authors:** Yui Sakai, Yen Yen Sally Rahayu, Tetsuya Araki

**Affiliations:** 1 Department of Global Agricultural Sciences, Graduate School of Agricultural and Life Science, The University of Tokyo, 1-1-1 Yayoi, Bunkyo Ward, Tokyo 113-8657, Japan; yui-sakai@g.ecc.u-tokyo.ac.jp; 2 Tokyo College, The University of Tokyo, 7-3-1 Hongo, Bunkyo Ward, Tokyo 113-0033, Japan; yenyenrahayu@gmail.com

**Keywords:** dietary habits, dietary intakes, canteen menus, university students, Indonesia

## Abstract

A comprehensive assessment of the dietary status of university students in Indonesia is lacking. Hence, this study aims to assess students’ dietary habits, status, and the nutritive value of meals offered at university canteens. This was a cross-sectional study based on the dietary habits of 333 students, 26 of whom were interviewed for the dietary survey. The nutritional value of canteen menus used by nearly half of the students (44%) was also examined. Most menus lacked macro and micronutrients (i.e., calcium, 15.5%) and were high in salt (181.5%). BMIs showed malnutrition among students (38.5%). The protein, fat, carbohydrate (PFC) ratio showed a high proportion of fat (32.4%) in the diets of female students. The level of salt intake (96.2%) was above the Indonesian recommended dietary allowance (RDA). Most students had unhealthy dietary patterns, including a high consumption of sweet beverages and instant noodles and a low intake of fruits, vegetables, animal protein, and milk. The lack of nutrients in canteen menus might lead to a nutrient deficiency among the students, which underlines the important role of canteens in the students’ dietary intake. Optimizing the nutritional profile of menus, labeling based on nutrient profiling, and promoting nutrition education should be addressed to improve students’ diets.

## 1. Introduction

Poor diet is linked to various non-communicable diseases (NCDs) and is potentially a significant contributor to NCD mortality globally [1,2]. Nutritional issues increase susceptibility to various diseases, including poor immunity against infections and NCDs, such as cardiovascular diseases, cancers, chronic respiratory diseases, and diabetes mellitus [3,4]. A review of 195 countries reported that 11 million deaths and 255 million disability-adjusted life-years (DALYs) were attributable to dietary risks, with a high intake of sodium and low intake of whole grains and fruits being the leading factors [5]. In Indonesia, dietary risks are also the leading factors responsible for the greatest disease burden, accounting for approximately 10% of the total DALYs [6]. The prevalence of NCDs has been increasing, not only among the elderly but also among young adults in Indonesia [7,8]. Similarly, to many other developing countries, particularly in Asia [9,10], Indonesia is also experiencing a double burden of malnutrition, with undernutrition and overnutrition seen in children and adults [7]. At least half of the Indonesian population suffers from at least one micronutrient deficiency [11], and one in seven adult women suffer from chronic energy deficiency [7]. In contrast, one in three adults are overweight or obese. Given the potential impact of dietary risks on NCD mortality and morbidity, promoting healthy dietary practices has become essential for health policy and NCD prevention [11,12,13].

Many studies have suggested that improving dietary habits through nutrition-based interventions could be particularly effective if performed on younger people. Studies on health behaviors have indicated that the nutritional habits of young people significantly influence their health status in later adulthood [14,15,16]. For example, a previous study identified that people aged between 18 to 29 years are at the life stage in which behavioral risk factors can lead to the development of cardiovascular diseases [17]. In addition, previous research found that there is a significant decline in physical activity during the transition to university [18,19]. The educational period is critical for developing a healthy lifestyle, including a healthy diet and adequate physical activity [20]; therefore, the dietary habits of university students are particularly relevant. In general, college is the first time young adults live independently and are more responsible for their health behaviors and risks [20]. Many studies have reported that to most students, nutritional concerns were less relevant than convenience [21,22,23]. Health behaviors were different among university students [20] and were associated with personal beliefs, emphasizing the importance of enhancing positive attitudes toward healthier lifestyles [24].

Out-of-home eating has significantly increased and has taken a prominent position in dietary habits over the past few decades [25,26]. Various studies have shown that out-of-home eating is associated with higher energy intake owing to its higher energy density or larger portions [27,28,29,30,31,32,33]. Psychosocial and environmental factors influence what is eaten, and consumers frequently lack access to the nutritional information that allows them to make an informed choice [34]. Compared with eating at home or self-catering, out-of-home eating also presents additional nutritional challenges, such as lower micronutrients, especially vitamin C, calcium, and iron [12]. Other reports have shown that a higher frequency of consuming meals prepared away from home was associated with a lower intake of healthy foods, such as fruits, vegetables, dietary fiber, vitamin C, minerals, a higher intake of fat and oils, and increased body weight and body mass index (BMI) [35].

Many students leave their family environment when entering university and move to university towns; therefore, university canteens have become a vital ‘out-of-home’ contributor in terms of where students consume their main meals [12]. A survey of university students in central Java indicated that more than two-thirds of the students had meals prepared away from home [36]; however, the study did not specify the whereabouts of out-of-home eating. Although no specific guidelines are issued for nutrition content in a school meal in Indonesia, the Indonesian dietary recommendations for a meal for young adults suggest 18.7–20.7 g protein, 25–30.3 g fat, and 103–125 g carbohydrates [7]. In addition, it advises that the total number of daily meals should cater for a minimum of five servings of vegetables and a maximum intake of 2 g of salt [7,37].

Although school canteens can contribute to creating dietary risks [38,39], they also represent an opportunity to improve students’ diets [40,41]. Several studies have investigated dietary habits and dietary status among university students in Indonesia [36,42,43,44,45,46,47,48]; however, these studies lack information regarding the nutritive value of the university canteen meals and the contribution of the food groups to students’ daily nutrient intake and food consumption. Thus, we aimed to assess students’ dietary habits, dietary status, and nutritive value of meals offered to them at the university canteen to fill the current literature gap. Our findings provide evidence-based information needed to set realistic goals for meal planning and develop nutrition-based intervention strategies to improve students’ health status and quality of life.

## 2. Materials and Methods

### 2.1. Study Design

A cross-sectional study was conducted among students at the Institut Pertanian Bogor (IPB), Indonesia, for one month between September and December 2019. Ethical approval was obtained from the Health Research Ethics Commission of the University of Tokyo (H-190905001).

Information on students’ dietary habits (dietary habit survey), including the sources and frequency of lunch meals on weekdays, was collected using an anonymous web-based survey from 917 students with 36.31% valid answers (*n* = 333). Respondents who received a questionnaire were excluded if they did not respond or did not answer all questions. The participants were randomly selected through student clubs and associations at the university. The meal sources in this study were categorized as follows: (1) university canteen, (2) eating outside, (3) self-catered/homemade, and (4) skipped meals. If the student had meals (canteen, eating outside except for canteen, self-catered, or skipped meals) at least twice in a weekday, it was counted as the student’s meal source. The frequency of meal sources counted was then calculated as a percentage by sex.

The nutritional value of the canteen menu was evaluated by measuring the weight and salinity of 20 types of meals served by the food stalls in the canteen. Among 333 students who participated in the survey on dietary habits, 100 were randomly selected to participate in the dietary intake assessment.

### 2.2. Data Collection

#### 2.2.1. Students’ Dietary Habits and Nutritive Value of Canteen Menu

Data on the students’ basic characteristics (i.e., gender, age, and faculty) and dietary habits were collected during the mid-academic term. The meals considered in this study were lunch meals due to the operating time of the university canteen from 11:00 a.m. to 4:00 p.m. In this study, the definition of a ‘canteen’ was an eating facility located in the university, comprising eighteen food stalls offering various menus on weekdays.

The students were considered to have a meal if they consumed solid food two or more times during weekdays, which produced energy. A self-catered meal was defined as a self-prepared or homemade meal. In contrast, a ‘prepared away-from-home’ or ‘out-of-home’ meal was defined as non-homemade food or a meal prepared outside of the home. Out-of-home eating included eating take-out food and eating out at restaurants, canteens, or other eating places. The definition of skipping a meal was self-explanatory.

The menu served by the food stalls in the canteen consisted of main and side dishes. Information on the ingredients, including the average weight of each ingredient, was collected twice on different days. The quantity of each ingredient was measured using a standard kitchen weight scale (Excellent Scale Co., Ltd., Jakarta, Indonesia). A salt meter (PAL-sio, ATAGO Co., Ltd., Tokyo, Japan) was used to measure the salt content of the menu, with an absolute measurement error of ±0.05% for 0.00–0.99% salt content and a relative measurement error of ±5% for 1.00–10.0% salt content [49]. Information on other seasonings, such as oils, was obtained from the cooks of the canteen’s food stalls. Food composition data (i.e., energy, carbohydrate, protein, fat, and sodium) were specified by referring to various databases, such as the Indonesian Food Composition Table 2005 [50], the USDA National Nutrient Database for Standard Reference 2015 [51], and the reference database of fat absorption of food processed by frying and moisture content changes due to the cooking process [52].

#### 2.2.2. Students’ Dietary Intakes

Students’ heights and weights were measured at the university using standardized protocols. BMI was used as an indicator of students’ nutritional status and was obtained by calculating the ratio of body weight (kg) to height squared (m) (expressed as kg/m^2^). Students’ nutritional status was categorized according to the WHO recommendations for underweight (BMI < 18.5), normal weight (BMI: 18.5–24.9), and overweight (BMI ≥ 25) [53]. The weekly hours of physical activity were asked. Physical activity was defined as moderate intensity aerobic physical activity according to WHO guidelines [54].

Of 333 students who participated in the dietary habit survey, 100 students were randomly selected to participate in the dietary intake survey. The dietary intake survey was conducted by administering questionnaires during a five-day observation. Among the 100 students receiving the questionnaires, some students who did not complete questionnaires for five days of observation were excluded. Their responses were considered invalid. Those whose responses were completed for five days of observation (valid responses) were included as a subsample (n = 26; 16 men and 10 women; 26.0% valid responses). Dietary intake was analyzed through a five-day observation of a subsample. The observed students were asked what they ate on five consecutive weekdays. All questionnaires were checked for completeness and errors on the same day or the day after administration. Missing data were collected by asking the students the same questions again. Food content was estimated by referring to the Indonesian Food Composition Table 2005 [50] and Buku Foto Makanan [55]. When the content of an item was not available in these databases, it was estimated by referring to recipes commonly used by the locals. Data on students’ food consumption were expressed in grams.

### 2.3. Dietary Analysis

The Indonesian Food Composition Table 2005 [50] and USDA National Nutrient Database for Standard Reference 2015 [51] were used for nutritional value calculations. Nutritional values were calculated using the following formula:

Nutritional value of ingredients = Σ [(Observed ingredient weight (g)
× Rate of weight changes (%) ÷ 100)
× Nutritional value per 100 g of ingredient weight (g)](1)

The amount of oil used was determined using Daftar Faktor Konversi Berat Bahan Makanan [52]. The following formula is used to determine the amount of oil used:

Oil weight (g) = (Raw ingredients weight (g) × Rate of oil used (%) ÷100)(2)

The Recommended Dietary Allowances (RDA) for Indonesia (Angka Kecukupan Gizi/AKG) [56] were used to evaluate whether the dietary intakes met the recommended amounts. The RDA used in this study were the recommended values for ages 19–29 based on sex. According to the U.S. government, one-third of the daily nutritional intake should be consumed for lunch; therefore, the nutritional intake per meal was set at one-third of the RDA [57].

Subsequently, detailed information regarding the intake of energy, protein, carbohydrate, fat, fiber, vitamins, minerals, and food groups (i.e., rice, vegetables, fruits, dairy, and chicken) was specified, and the contributors to the intake of each nutrient by different food groups were obtained. Each ingredient was assigned to a food group to assess its contribution to the total nutrient intake. The sources of each nutrient were listed in descending order of the percentage of nutrient intake for each source to the fifth largest source.

Data were expressed as the mean ± SD and were analyzed statistically using Python software version 3.8. Repeated–measures analysis of variance (ANOVA) was used to compare means among the number of students who had self-catered meals, prepared away from home meals, and skipped meals. It was also used to compare means of the amount of intake of each food group by sex. When the weekly hours of physical activity were found to be significantly associated with BMI, the difference was analyzed using Student’s *t*-test. Differences were considered statistically significant at *p* > 0.05.

## 3. Results and Discussion

### 3.1. The Students’ Dietary Habits

Table 1 shows that significantly more students had meals prepared away from home than self-catered meals on weekdays (eating out and taking out). Details of the frequency and its percentage value are shown in Supplementary Table S1. Similar to previous studies [58,59], the most common reason for having meals prepared away from home was the living conditions of most students: living alone without family. Among the students who had meals prepared away from home, just over half had meals in the canteen. This result suggests that the canteen menu may play a major role in students’ dietary intake.

Evaluation of the canteen menu (Table 2) indicated that the nutritional values of most menus were deficient. Generally, compared with a self-catered meal, the nutritional value of meals prepared away from home, including a school meal, tends to be lower in micronutrients, especially vitamin C, calcium, and iron [12,35]. In addition, we found that the average salt content of the canteen menu items was almost twice the RDA. Previous studies reported that meals prepared outside of the home, such as on the streets, in restaurants, and fast food, were the major contributors to salt intake [60,61]. Considering that consuming meals in the canteen is a common practice, there is a possibility of nutrient deficiency among students. To confirm this, we analyzed the dietary intake of the students.

### 3.2. Dietary Intakes

Dietary intakes were analyzed through a five-day observation of a subsample (*n* = 26; 16 man and 10 woman) to assess their dietary status.

#### 3.2.1. BMI Status

Poor nutrition or nutritional imbalance is measured using weight indicators; such imbalance results in either being underweight, overweight, or obese [62]; therefore, we measured the BMI of the observed students. Table 3 shows that almost 40% of the observed students had poor nutritional status: underweight (15%) and overweight (23%). Nutritional studies among students in other developing Asian countries have shown similar trends. For example, in Thailand, 21.9% and 9.8% of students are underweight and overweight, respectively [63]. A survey among female university students in Bangladesh also reported a prevalence of an underweight status at 23.9%, an overweight and obese status at 9.10%, and a normal nutritional status at 70.0% [64]. These reports and our observations confirm the double burden of malnutrition, where both underweight and overweight statuses exist in the same population, household, or even a fluctuating individual [65]. Although high-income countries tend to have a higher prevalence of overweight people and low-income countries have a higher prevalence of underweight people, the double burden of malnutrition is most prevalent in middle-income countries, such as Kyrgyzstan, Russia, and Indonesia [9]. The double burden of malnutrition is receiving greater attention as it appears to be more permanent and widespread than previously perceived, with a particular rise in Asia [10].

#### 3.2.2. Daily Dietary Intake

Table 4 presents the daily nutrient intake, including energy, protein, fat, carbohydrate, fiber, minerals, vitamins, and salt, for the observed students. Generally, most of them did not meet the dietary requirements for many key macronutrients and micronutrients. Except for sodium and vitamin K, none of the micronutrients, such as fiber, potassium, iron, and calcium, were adequate in the students’ dietary intake.

- *Energy intake, weight status and physical activity*

The observed mean energy intakes (kcal/capita/day) was 1693.3 ± 348.5 for male students and 1393.0 ± 294.2 for female students, showing a tendency for energy intake to be below the RDA. In a previous study of female college students, energy intake was 1429.8 ± 516.7 kcal, and many of them did not exceed the RDA [66]. In the present study, 23.1% of the observed students were overweight, even though they were below the RDA for energy. Similar trends have been reported in many studies of university students. For example, in a study of Turkish students, 20% were overweight despite an energy intake of 80% of the RDA [67]. Another study conducted in Spain showed that 20% of students were overweight even though their energy was below the RDA [68].

**Figure 1:**
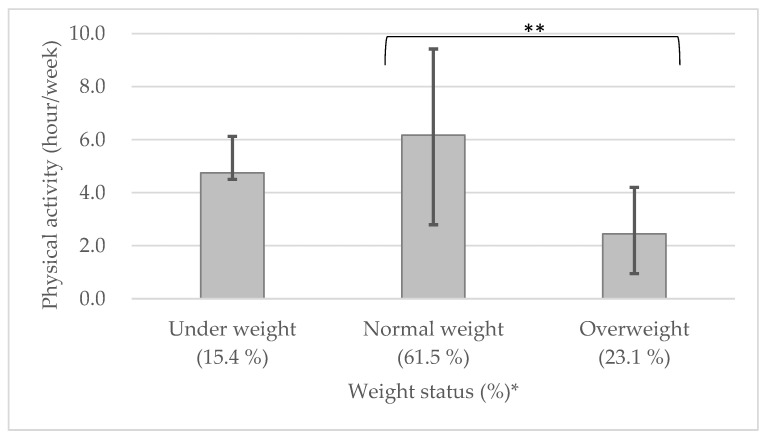
Association between physical activity and body weight of observed students. * Based on body mass index (BMI; in kg/m2). Under 18.5: underweight, over 18.5 and under 25.0: normal weight and over 25.0: overweight. ** *p* < 0.05: weight status vs. the weekly hours of physical activity. Significant differences correspond to Student’s t-test with each category of weight status.

The percentage of overweight students was 23.1% even though the energy intake was below the RDA. Other than energy intake from food consumption, the percentage of overweight students may also be associated with other factors, such as the amount of physical activity. This study showed that overweight students were significantly less physically active than normal weight students (Figure 1). A previous study reported that physical activity and body weight are correlated; increases in inactivity are associated with increases in weight [69]. Another study showed that despite equal average daily energy intakes, BMIs were 1.7% higher in men and 8.3% higher in women with low physical activity than those with ideal physical activity levels [70]. These results indicated that the amount of physical activity significantly impacted BMI.

Considering that consumption varies with the amount of physical activity, and that energy intake is below the RDA despite the high percentage of overweight students, there may be a need to reevaluate the RDA. Prior research pointed out that although increased use of technology has reduced the need for energy consumption [71,72], there has been no systematic reevaluation of recommendations regarding energy intake [73]. Furthermore, the decrease in energy consumption has not been fully compensated for by increased spontaneous physical activity [73]. Recommendations regarding energy intake need to be reevaluated to consider individual differences in activity levels [73].

According to the level of consumption (Table 5), rice was one of the main contributors of calorie intake (30.6–36.3%). In addition, instant noodles (8.7–14.0%), oil and fats (10.1–12.0%), and chicken (4.7–5.1%) were found to be the food groups that also contributed to the daily energy intake. The upper end of the graph describes the third quartile, and the lower end of the graph describes the first quartile.

b. *Protein, fat, and carbohydrate (PFC) ratio*

**Figure 2:**
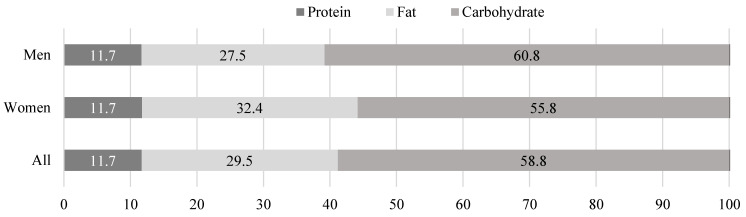
Contribution of protein, fat, and carbohydrate to the total energy intake (%).

Further analysis of the energy ratio from PFC indicated that carbohydrates (58.8%) mainly contributed to the total energy (Figure 2). According to previous research, the PFC ratio of Indonesians shifted from 10:8:82 in 1983 to 18:28:54 in 2004, indicating that carbohydrates still constitute the greatest proportion of energy, but to a lesser extent [74]. In contrast, protein constituted the lowest proportion of the PFC ratio, with a mean intake of 11.7%. Table 5 shows that the main protein source for most observed students was grains rather than animal proteins [37,75]. The RDA suggests that 55–60% of energy should come from carbohydrates and 10–20% from proteins [56]. Thus, the proportion of carbohydrates and proteins in the present study was within the recommended levels. Although we found that the average fat proportion in the PFC ratio (29.5%) was lower than that in the RDA, the fat proportion for female students (32.4%) was greater than the recommended amount of 30% or less [56]. Similarly, previous research found that the proportion of lipids was often predominant in the PFC ratio among Indonesian women, and has increased over the years [74]. This finding is also in line with other studies showing that total calories from fat were higher in women than in men [66,76,77]. This may be due to a lower carbohydrate intake compared with male students. In a previous study, the authors explained that female students’ proportion of lipids was higher than males’ because of their lower carbohydrate intake [66].

c. *Salt intake*

This study found that the daily salt intake was above the recommended amount for 96.2% of the observed students (4.8 to 6.4 g). The WHO recommends a daily salt intake of less than 5 g (approximately 2 g sodium) in adults (≥16 years) to reduce blood pressure and the risk of cardiovascular disease, stroke, and coronary heart disease. Indonesia has the highest prevalence of hypertension compared with other Southeast Asian countries such as Malaysia, Singapore, Thailand, the Philippines, and Vietnam [78]. Furthermore, the risk of hypertension appears to be greater for Indonesians than for Chinese and Vietnamese individuals with the same BMI [79]. In line with previous Indonesian studies [37,80], the sodium intake of male students tended to be higher than that of female students. Although a previous study reported that the highest salt intake among Indonesian adults was attributed to cereal and cereal product consumption [37], in this study, we found that seasoning was the major contributor to the total salt intake of the observed students (Table 5). Other food groups that contributed to total salt intake included eggs, dairy, noodles, bread, beef, and snacks.

d. *Dietary intake from canteen menu*

The daily intake of energy and nutrients of the observed students from canteen meals ranged from 14.1% to 28.2% (Table 4). Consumption of more than 25% of total daily energy intake at locations other than households was considered to be substantial out-of-home eating [26]. Thus, it is safe to suggest that the dietary intake status of the students in this study depends on the meal provided in the university canteen. Moreover, our findings confirm the possibility of nutrient deficiency among the students, as the nutritional content of the canteen menu was below the RDA per meal (Table 2). These findings point toward the necessity for improving the nutritional content of the canteen menu.

#### 3.2.3. Food Consumption Pattern

Lower nutrient intake is generally associated with a low consumption of healthy foods, such as fruits and vegetables, and a higher intake of oils and fats [35]; therefore, we examined the food consumption patterns of the observed students (Table 6).

Table 6 shows that the total daily food consumption of observed students was estimated as 957.1 g/capita/day: 1039.2 for men and 856.8 g/capita/day for women. Generally, rice, vegetables, and milk were the main contributors to the daily food consumption of both men and women. Sweet beverages were the second-highest food group, contributing to the total average consumption by 2.96–11.29%, but the consumption was significantly higher in men. In contrast, fruits, which were among the top five food groups, were consumed more by the female students, representing a 3.60–10.13% contribution to the total food consumption, but the difference was not statistically significant.

e. *Rice (cereals) and instant noodles consumption*

Average rice consumption (g/day) was 327.9 ± 118.7 (Table 5). This study found that the average daily rice consumption of the students was comparable to those of previous case studies among adults living in the capital city of Jakarta [37] and the Padang city of West Sumatera [81], but higher than that of the Indonesian population (~265 g/day) [82]. Generally, rice consumption in Indonesia has decreased over the last three decades [82,83]. As shown in Table 5, rice products are the main source of dietary fiber, and many vitamins and minerals such as calcium and iron [84]. The current declining trend in rice consumption implies a possible deficiency in these essential nutrients.

According to previous Indonesian studies, the trend of declining rice consumption is partly due to the increasing consumption of instant noodles [85,86]. Accordingly, we found that noodles and instant noodles were among the students’ top ten most consumed food groups. With an average consumption (g/day) of 51.0 ± 73.8 and 33.2 ± 39.4, noodles and instant noodles represent 2.45–7.26% and 2.26–5.28% of total consumption, respectively. Notably, male students consumed more noodles whereas female students consumed more instant noodles. Instant noodle consumption is relatively high among the Asian population, making this food group one of the main sources of total energy intake and carbohydrates, followed by rice, and sources of fat [87,88,89]. Similarly, in the present study, instant noodles contributed to 8.7–14% of the total energy intake and 8.7–15.3% of the carbohydrate intake of the observed students, second only to rice. We also found that the instant noodles food group was the second most common fat source after the oils and fats group. This finding may explain the fat disproportion in the PFC ratio among the observed female students.

Although the contribution of instant noodle consumption to increasing health risk factors remains debatable [90,91], a survey on health and nutrition reported an increased prevalence of cardiometabolic risk factors associated with a high intake of Western fast foods and instant noodles [92]. Later in life, instant noodle consumption alone might contribute to higher cardiometabolic risk and abdominal obesity in women, independent of overall dietary patterns [88,92]. Many studies have also found a high salt content in the seasoning of instant noodles [87,88,89]. Noodles, the third most consumed food group among male students, consisted of noodle dishes that contained excessive seasonings, such as *mie bakso* (noodle with meatball soup) and *mie ayam* (chicken noodle) [89,93,94]. This suggests that students’ consumption of noodles and instant noodles may lead to increased seasoning consumption, and thus, to higher salt intake.

f. *Consumption of sweet beverages*

Sugar consumption observed in this study ranged from 6.8 to 14.4 g/day. These values were significantly lower than the daily intake of young adults in the US, which reached 66.8–94 g/day [95]. In the present study, the students’ sugar intake was lower than the Indonesian average of 25.6 g/day [37]; however, this difference may be due to our calculation of sugar consumption, which excluded sugar content from sweet beverages and snacks. Many studies have reported that high sugar intake was attributed to the high consumption of sweetened or carbonated beverages [37,80,96]. A recent global study also reported that beverages were the main contributor to total sugar intake, with an average intake of 49 g/day from sugar-sweetened beverages, which is far higher than the optimal intake level of 3 g/day [5]. It should be noted that the students’ average consumption of the sweet beverages group (g/day) was 76.1 ± 85.2, exceeding the global average, and making it the second most consumed food group after rice (2.96–11.29%). Considering the sugar content in sweet beverages, the actual total sugar intake in the present study may be higher than the average of 11 g/day, suggesting that the sugar intake might be high, particularly among male students. Excessive sugar consumption has been associated with obesity and cardiovascular diseases [97,98,99,100]. In this regard, a reduction in free sugar intake to 25–37.5 g/day (6–9 teaspoons) has been advised [100]. The WHO also recommends that free sugar intake be less than 10% of the total energy intake or 50 g/day (12.5 teaspoons) to prevent health risks [101].

g. *Consumption of poultry products, fruits, vegetables, and milk*

The observed students consumed various animal proteins, with chicken (45.8 g/day) and eggs (42.9 g/day) identified as the major sources at an average of 9.27% of the total food consumption, followed by beef (21.9 g/day), and fish (19.0 g/day) (4.36% contribution); however, as shown in Table 4, the average total protein intake (45.6 g/day) was below the recommended amount of 57 g/day [82], which might partially explain the high prevalence of micronutrient inadequacies among the observed students. Low protein intake, particularly from animal proteins, has been associated with a deficiency of blood-forming nutrients, such as iron, folic acid, vitamin B_6_, and vitamin B_12_ [102]. A previous study reported that animal proteins contain highly bioavailable heme iron and can increase non-heme iron absorption [103]. An insufficient amount of these micronutrients may lead to an increased risk of anemia [104], heart disease [105], or aging-related cognitive problems [106,107]. In Indonesia, iron-deficiency anemia is one of the top five leading causes of years lived with disability (YLDs) [6].

An increased intake of vegetables and fruits can compensate for the deficit in animal protein [108]. Diets with a high consumption of vegetables and fruits have also been identified to prevent NCDs risks [5]. Nevertheless, we also found that the students’ intake of vegetables and fruits was inadequate, regardless of the most consumed food groups. Although the health benefits of fruit and vegetable consumption are well known [109], and a considerable amount of work has been attempted to improve their consumption, the intake remains low globally [108,110]. The present study estimated the students’ consumption of vegetables and fruits (g/day) as 62.6 ± 36.3 and 59.5 ± 84.2, respectively, which is 20% less than the RDA level for Indonesian adults [56]. Our finding is in line with a national survey that reported low consumption of vegetables and fruits among the Indonesian population [111]. A global review revealed that interventions to increase fruit and vegetable intake more often targeted fruits, and typically reported greater success in fruit consumption than in vegetables [112]. As a consequence, vegetable intake is lower than that of fruits, regardless of the improved health benefits from high consumption due to their protein and fiber content [109]. Furthermore, most interventions aimed at increasing vegetable intake in Indonesia and elsewhere have focused on younger children [112,113].

The students’ average milk consumption (g/day) was estimated at 62.5 ± 108.6, which is higher than that of the Indonesian population [82]. Milk contributes to the supply of calcium, vitamin A, and vitamin D [15,114]; thus, the low calcium intake of the observed students may be attributed to their low milk consumption. In the last decade, Indonesian milk consumption has increased, which is attributed to the efforts of the Indonesian government to promote its consumption among students [113]; however, the average consumption is still lower than that of students in other countries [66,75,77,114]. According to a previous report, the milk intake of Indonesian adults aged 25 and older was lower than that of Asians and the global average [8].

## 4. Conclusions and Recommendations

This study demonstrated that most university students had lunch meals prepared away from home, with just over half of them having it in the university canteen. Given the importance of lunch as a main meal of the day, this finding suggests that the university canteens’ menus may significantly influence students’ dietary intake; however, it was found that the nutritional value of most menus was deficient in both macro- and micronutrients, and high in sodium and salt content, at almost twice that of the RDA. The BMI indicator showed the problem of the double burden of malnutrition among the observed students.

Further examination of the daily nutrient intake indicated that none of the observed students met the recommended daily nutrient intake. Except for sodium and vitamin K, none of the micronutrients, such as fiber, potassium, and iron, were taken adequately. Male students tended to have a higher energy intake than female students, with rice as the main contributor to the calorie intake (30.6–36.3%), followed by instant noodles (8.7–14.0%), oil and fats (10.1–12.0%), and chicken (4.7–5.1%). Further analysis of the energy ratio from PFC indicated that carbohydrates mainly contributed to the total energy, whereas protein constituted the lowest in the PFC ratio. We also found that the fat proportion of the PFC ratio among female students was higher than that of male students, and the mean total calories from fat for women was greater than the recommended amount of 30% or less. The amount of salt intake was above the RDA level, with seasoning being a major contributor to the total salt intake of the observed students. Other food groups that contributed to the total salt intake included eggs, dairy, noodles, bread, beef, and snacks. The food consumption evaluation demonstrated that most students had unhealthy eating patterns, including a high consumption of sweet beverages, instant noodles, seasonings, snacks, and a low intake of fruits, vegetables, animal proteins, legumes, and milk.

Findings from this study indicated that the lack of nutrients in most menus of university canteens might lead to nutrient deficiency among the students. Given the important role of university canteens as a contributor to the consumption of the main meal prepared away from home (i.e., lunch), optimizing the nutritional profile of canteen menus opens a window of opportunity to improve students’ diets. For example, serving a cooked meal with more vegetables during lunchtime is an efficient way to improve the diet of canteen customers [115]. A reduction in salt intake from the canteen menu can be achieved by gradually reducing the amount of salt added to foods by the cooks. Furthermore, labeling based on nutrient profiling is a promising way to introduce an informed choice among students [116], thus instigating healthy choices of food items [117]. Awareness-building, health-promoting campaigns are also needed to encourage students to consume more vegetables and fruits, and less food with a high salt content. Moreover, although the average sugar consumption of students was still lower than the optimum value set by the WHO, reducing its intake could be associated with improved health conditions. Considering that beverages were identified as the main contributors to sugar intake, it is sensible to advocate for students, particularly men, to minimize the consumption of beverages with added sugars. Furthermore, in addition to nutrition education, programs that support physical activity need to be introduced. Health education should work with campus officials to develop an environment that stimulates physical activity. For example, by closing the campus to vehicular traffic during the day, adding wide sidewalks, and building walking and bicycle pathways. Finally, universities provide opportunities to positively influence many young adults’ nutrition and healthy behaviors in an educational setting. Thus, efforts toward nutrition improvement should be accompanied by increasing efforts to provide well-planned advocacy that incorporates nutrition education in the academic setting.

## Figures and Tables

**Table 1:** The frequency of students’ dietary habits on five consecutive weekdays (*n* = 333).

Meal Sources	Frequency (%)			*p* Value
	Men	Women	All	
Self-catered	13	9	11	
Prepared away from home				
Canteen	53	32	44	*
Eating outside	29	49	37	*
Skipped meals	6	10	8	

Gender difference for each variable was analyzed using ANOVA and the asterisks represent a *p* value < 0.05 that is considered statistically significant.

**Table 2:** The mean nutrition content of the canteen menus.

Nutrients	Mean ± SD	RDA per Meal *	
		Men	Women
Energy (kcal)	539.0 ± 128.6	908	750
Protein (g)	17.9 ± 6.0	20.7	18.7
Fat (g)	13.7 ± 7.6	30.3	25.0
Carbohydrate (g)	80.2 ± 19.9	125.0	103.0
Fiber (g)	3.7 ± 3.8	12.7	10.7
Sodium (mg)	924.1 ± 360.8	500	500
Potassium (mg)	415.0 ± 266.1	1567	1567
Calcium (mg)	57.0 ± 31.8	367	367
Magnesium (mg)	51.0 ± 39.6	117	103
Phosphorus (mg)	221.6 ± 82.7	233.3	233.3
Iron (mg)	1.8 ± 1.0	4.3	8.7
Zinc (mg)	2.2 ± 0.8	4.3	3.3
Copper (mg)	0.3 ± 0.2	300	300
Manganese(mg)	0.8 ± 0.3	0.8	0.6
Vitamin A (μg)	99.4 ± 89.3	200	167
Vitamin D (μg)	0.7 ± 0.8	5.0	5.0
Vitamin E (mg)	2.0 ± 1.4	5.0	5.0
Vitamin K (μg)	36.8 ± 20.52	21.7	18.3
Vitamin B_1_ (mg)	0.1 ± 0.1	0.47	0.37
Vitamin B_2_ (mg)	0.2 ± 0.1	0.53	0.47
Niacin (mg)	3.1 ± 2.2	5	4
Vitamin B_6_ (mg)	0.3 ± 0.2	0.4	0.4
Vitamin B_12_ (μg)	0.8 ± 1.9	0.8	0.8
Folic acid (μg)	44.5 ± 22.7	133.3	133.3
Vitamin C (mg)	7.6 ± 5.8	30	25
Salt (g)	2.4 ± 0.9	1.3	1.3

* Recommended Daily Allowance (Angka Kecukupan Gizi/AKG) [56].

**Table 3:** Basic characteristics of students.

Gender (%)		Height	Weight	Average of BMI	Weight status (%) *			PA **
		(cm)	(kg)		Under Weight	Normal Weight	Over-Weight	
Men	61.5	169.5 ± 4.4	67.2 ± 14.2	23.2 ± 4.3	18.8	43.8	37.5	5.9
Women	38.5	157.7 ± 6.0	52.4 ± 5.8	21.1 ± 2.4	10.0	90.0	0.0	3.9
All		165.0 ± 7.5	61.5 ± 13.4	22.4 ± 3.6	15.4	61.5	23.1	5.1

* Based on body mass index (BMI; in kg/m^2^). Under 18.5: underweight, over 18.5 and under 25.0: normal weight and over 25.0: overweight. ** The weekly hours of physical activity.

**Table 4:** The mean daily nutrient intake and the percentage of students who met the RDA.

Nutrients	Men	Women	All	Daily Nutrients Intake from Canteen Meals (%) *	Students Met the RDA ** (%)
Energy (kcal)	1693.3 ± 348.5	1393.0 ± 294.2	1558.7 ± 357.9	24.2	0.0
Protein (g)	49.4 ± 11.6	40.9 ± 7.2	45.6 ± 10.7	21.4	11.5
Fat (g)	51.7 ± 13.7	50.1 ± 14.3	51.0 ± 14.0	24.2	3.8
Carbohydrate (g)	245.5 ± 52.8	186.5 ± 37.4	219.1 ± 55.0	24.4	0.0
Fiber (g)	9.3 ± 4.1	7.2 ± 2.5	8.4 ± 3.6	20.6	0.0
Sodium (mg)	2536.1 ± 912.2	1922.7 ± 623.8	2261.1 ± 852.4	23.4	96.2
Potassium (mg)	1250.3 ± 387.7	1068.5 ± 277.9	1168.8 ± 354.6	21.2	0.0
Calcium (mg)	219.5 ± 99.0	221.0 ± 104.2	220.2 ± 101.4	20.7	0.0
Magnesium (mg)	132.5 ± 49.6	110.9 ± 34.6	122.8 ± 44.8	24.1	0.0
Phosphorus (mg)	618.3 ± 163.3	526.8 ± 131.3	577.3 ± 156.5	23.0	23.1
Iron (mg)	4.7 ± 1.2	3.6 ± 0.9	4.2 ± 1.2	23.8	0.0
Zinc (mg)	6.2 ± 1.4	4.5 ± 1.1	5.4 ± 1.5	24.7	0.0
Copper (mg)	0.8 ± 0.2	0.6 ± 0.1	0.7 ± 0.2	28.2	0.0
Manganese(mg)	2.2 ± 0.5	1.6 ± 0.5	1.9 ± 0.6	28.1	38.5
Vitamin A (μg)	269.4 ± 187.9	249.1 ± 157.1	260.3 ± 175	17.6	7.7
Vitamin D (μg)	1.9 ± 1.6	2.0 ± 2.4	2.0 ± 2.0	21.4	0.0
Vitamin E (mg)	5.6 ± 1.7	5.2 ± 1.0	5.4 ± 1.4	25.2	0.0
Vitamin K (μg)	91.6 ± 25.4	79.2 ± 24.6	86.0 ± 25.8	23.7	88.5
Vitamin B_1_ (mg)	0.4 ± 0.1	0.3 ± 0.1	0.3 ± 0.1	28.4	0.0
Vitamin B_2_ (mg)	0.6 ± 0.1	0.5 ± 0.1	0.5 ± 0.1	27.0	0.0
Niacin (mg)	8.1 ± 2.6	6.9 ± 1.7	7.6 ± 2.3	19.9	0.0
Vitamin B_6_ (mg)	0.6 ± 0.1	0.5 ± 0.1	0.6 ± 0.1	22.3	0.0
Vitamin B_12_ (μg)	2.5 ± 2.4	3.2 ± 2.2	2.9 ± 2.3	14.1	50.0
Folic acid (μg)	123.7 ± 43.5	103.7 ± 27.7	114.8 ± 38.6	20.5	0.0
Vitamin C (mg)	29.2 ± 18.3	37.5 ± 20.1	32.9 ± 19.6	19.0	3.8
Salt (g)	6.4 ± 2.3	4.8 ± 1.5	5.7 ± 2.1	23.4	96.2

* The percentage of daily nutrient intake from canteen meals is relative to the total daily intake. ** Recommended Daily Allowance (Angka Kecukupan Gizi/AKG) [53].

**Table 5:** The main contributors to the intake of each nutrient by different food groups.

**Energy**				**Protein**				**Fat**			
**Men**	**(%)**	**Women**	**(%)**	**Men**	**(%)**	**Women**	**(%)**	**Men**	**(%)**	**Women**	**(%)**
Rice	36.3	Rice	30.6	Rice	19.1	Chicken	21.0	Oils and Fats	38.3	Oils and Fats	37.6
Oils and Fats	10.1	Instant Noodles	14.0	Chicken	18.8	Rice	15.5	Instant Noodles	14.5	Instant Noodles	11.0
Instant Noodles	8.7	Oils and Fats	12.0	Eggs	11.8	Instant Noodles	9.6	Chicken	8.0	Eggs	9.6
Noodles	6.0	Chicken	5.1	Beef	9.4	Eggs	9.0	Eggs	6.5	Chicken	8.7
Chicken	4.7	Bread	4.4	Beans	6.6	Fish	8.7	Beef	6.1	Beef	5.2
**Carbohydrate**				**Fiber**				**Potassium**			
**Men**	**(%)**	**Women**	**(%)**	**Men**	**(%)**	**Women**	**(%)**	**Men**	**(%)**	**Women**	**(%)**
Rice	53.3	Rice	49.2	Vegetables	28.9	Vegetables	27.0	Vegetables	18.3	Vegetables	14.6
Instant Noodles	8.7	Instant Noodles	15.3	Beans	15.3	Instant Noodles	13.8	Chicken	12.5	Chicken	12.2
Noodles	7.8	Bread	6.3	Rice	14.4	Rice	13.0	Beans	10.0	Tubers	10.4
Sugar	5.4	Sweets and Snacks	4.2	Instant Noodles	9.3	Beans	11.3	Rice	9.2	Fruits	9.2
Tuber	4.7	Tubers	3.7	Noodles	9.0	Fruits	6.9	Fruits	7.9	Beans	7.5
**Calcium**				**Magnesium**				**Phosphorus**			
**Men**	**(%)**	**Women**	**(%)**	**Men**	**(%)**	**Women**	**(%)**	**Men**	**(%)**	**Women**	**(%)**
Dairy	17.8	Dairy	26.7	Rice	21.8	Beans	17.5	Rice	21.1	Chicken	16.5
Vegetables	15.4	Fish	16.9	Beans	17.3	Rice	17.2	Chicken	14.6	Rice	16.4
Fish	13.4	Vegetables	11.4	Chicken	9.0	Chicken	9.7	Eggs	13.9	Dairy	12.5
Eggs	12.3	Eggs	9.4	Vegetables	7.3	Fruits	7.5	Beans	7.9	Eggs	10.6
Beans	10.5	Beans	7.8	Seasonings	6.2	Tubers	6.2	Dairy	7.2	Fish	10.5
**Iron**				**Zinc**				**Vitamin A**			
**Men**	**(%)**	**Women**	**(%)**	**Men**	**(%)**	**Women**	**(%)**	**Men**	**(%)**	**Women**	**(%)**
Eggs	21.8	Eggs	17.7	Rice	36.8	Rice	33.7	Eggs	40.6	Eggs	28.0
Beef Meats	11.9	Chicken	11.2	Beef	16.2	Chicken	17.9	Chicken	16.0	Vegetables	19.2
Rice	11.4	Beef	11.1	Chicken	13.9	Beef	11.7	Vegetables	14.6	Dairy	18.5
Beans	11.2	Rice	10.4	Eggs	10.1	Eggs	8.6	Dairy	10.6	Chicken	12.7
Vegetables	10.8	Beans	9.8	Beans	5.5	Dairy	6.5	Sweets and Snacks	6.4	Sweets and Snacks	5.3
**Vitamin D**				**Vitamin E**				**Vitamin K**			
**Men**	**(%)**	**Women**	**(%)**	**Men**	**(%)**	**Women**	**(%)**	**Men**	**(%)**	**Women**	**(%)**
Eggs	51.1	Eggs	34.0	Oils and Fats	46.6	Oils and Fats	46.6	Eggs	51.1	Eggs	34.0
Fish	14.8	Fish	26.5	Eggs	10.2	Vegetables	7.4	Fish	14.8	Fish	26.5
Chicken	13.4	Chicken	15.1	Vegetables	9.4	Eggs	6.9	Chicken	13.4	Chicken	15.1
Dairy	6.8	Dairy	9.8	Chicken	6.2	Nuts	6.2	Dairy	6.8	Dairy	9.8
Mushrooms	5.5	Beef	4.6	Nuts	5.8	Fish	5.8	Mushrooms	5.5	Beef	4.6
**Vitamin B_1_**				**Vitamin B_2_**				**Folic acid**			
**Men**	**(%)**	**Women**	**(%)**	**Men**	**(%)**	**Women**	**(%)**	**Men**	**(%)**	**Women**	**(%)**
Rice	20.1	Rice	14.5	Eggs	31.5	Eggs	23.5	Vegetables	26.4	Vegetables	24.0
Chicken	13.6	Chicken	13.6	Chicken	13.7	Chicken	17.0	Eggs	16.8	Eggs	12.3
Vegetables	10.9	Beef	8.6	Dairy	9.3	Dairy	15.6	Rice	10.5	Fruits	11.4
Beef Meats	8.4	Vegetables	8.0	Beef	8.8	Vegetables	8.6	Beans	7.6	Tubers	8.6
Eggs	6.8	Nuts	7.9	Vegetables	7.8	Beef	6.3	Fruits	7.2	Rice	7.9
**Vitamin C**				**Salt (g)**							
**Men**	**(%)**	**Women**	**(%)**	**Men**	**(%)**	**Women**	**(%)**				
Vegetables	55.2	Vegetables	36.4	Seasonings	84.5	Seasonings	79.5				
Fruits	21.1	Fruits	29.1	Eggs	3.0	Dairy	3.0				
Tuber	9.0	Tubers	22.5	Noodles	2.9	Bread	2.7				
Chicken	6.8	Chicken	4.3	Snacks	2.2	Beef	2.6				
Seasonings	2.6	Beef	2.7	Bread	2.6	Eggs	2.5				

**Table 6:** Food consumption pattern of observed students.

No.	Food Group	Food Consumption (g/capita/day)		
		Men (Mean ± SD)	Women (Mean ± SD)	All (Mean ± SD)
1	* Rice	366.6 ± 126.6	280.2 ± 87.2	327.9 ± 118.7
2	Bread	15.3 ± 15.1	22.0 ± 19.6	18.3 ± 17.6
3	* Noodles	75.4 ± 88.0	21.0 ± 31.3	51.0 ± 73.8
4	Instant noodles	23.5 ± 23.9	45.2 ± 49.9	33.2 ± 39.4
5	Tubers	19.5 ± 18.4	31.1 ± 26.0	24.7 ± 22.9
6	* Sugar	14.4 ± 8.5	6.8 ± 5.1	11.0 ± 8.1
7	Beans	25.7 ± 33.2	21.3 ± 27.8	23.7 ± 30.9
8	Nuts	3.7 ± 6.7	4.5 ± 5.4	4.1 ± 6.2
9	* Vegetables	72.7 ± 41.6	50.2 ± 23.0	62.6 ± 36.3
10	Fruits	37.4 ± 38.0	86.8 ± 112.6	59.5 ± 84.2
11	Mushrooms	1.6 ± 4.9	1.7 ± 3.3	1.6 ± 4.3
12	Fish	14.5 ± 18.0	26.4 ± 33.1	19.8 ± 26.6
13	Beef	22.8 ± 18.9	20.9 ± 23.4	21.9 ± 21.0
14	Chicken	48.7 ± 25.9	42.9 ± 22.5	45.8 ± 43.9
15	Eggs	49.1 ± 35.3	35.2 ± 26.5	42.9 ± 32.4
16	Milk	61.1 ± 108.5	64.1 ± 108.8	62.5 ± 108.6
17	Oils and Fats	19.0 ± 9.4	20.8 ± 6.9	19.8 ± 8.4
18	Snacks and Sweets	22.7 ± 31.6	23.4 ± 32.5	23.0 ± 32.0
19	* Sweets beverages	117.3 ± 92.6	25.4 ± 31.4	76.1 ± 85.2
20	Seasonings	28.2 ± 15.0	26.9 ± 19.8	27.7 ± 17.3
Total		1039.2	856.8	957.1

* Gender difference for the intake levels of each food group were analyzed using ANOVA and the asterisk represents a *p* value < 0.05 that considered as statistically significant.

## Data Availability

Data will be made available upon request by author Yui Sakai.

## Supplementary Materials

The following supporting information can be downloaded at: https://www.mdpi.com/article/10.3390/nu14091911/s1, Table S1: The number and percentage of students’ meal pattern in five consecutive weekdays (*n* = 333).
